# Association of Hepatitis C Virus Infection and Interleukin-28B Gene Polymorphism in Chinese Children

**DOI:** 10.12669/pjms.303.4267

**Published:** 2014

**Authors:** Rong-Rong Wu, Feng-Qun Liu, Shi-Shu Zhu, Jin Han

**Affiliations:** 1 Rong-Rong Wu, Department of Pharmacy, Beijing 302 Hospital, Beijing China.; 2 Feng-Qun Liu, Department of Pharmacy, Beijing 302 Hospital, Beijing China.; 3 Shi-Shu Zhu, Department of Pediatric Hepatology, Beijing 302 Hospital, Beijing China.; 4 Jin Han, Department of Pharmacy, Beijing 302 Hospital, Beijing China.

**Keywords:** Hepatitis C virus, Interleukin-28B, Single nucleotide polymorphism

## Abstract

***Objective: ***To preliminarily explore the association of rs12979860 and rs8099917 SNPs with chronic HCV infection in Chinese Han children.

***Methods: ***Chronic HCV infection patients (n=277; 1-17 years old, 4.5 years old in average) and healthy subjects (n=150, children; 2-17 years old, 5.2 years old in average) were recruited and tested by PCR combining direct sequencing. The differences between the rs12979860 and rs8099917 genotypes in patients and healthy subjects were compared.

***Results:*** The genetic variations at rs12979860 and rs8099917 in chronic hepatitis C (CHC) children and healthy subjects did not differ significantly. The frequency of spontaneous clearance in CHC children was higher (47%), which is related to the genetic variations. The histological changes of patients were more significant compared to their clinical and biochemical indices, but they did not correlate with the genetic mutations at rs12979860 and rs8099917 significantly.

***Conclusion: ***The rs12979860 and rs8099917 SNPs are independent factors predicting the spontaneous clearance of Chinese CHC children patients. The correlation between diseases outcomes are in need of further study.

## INTRODUCTION

Hepatitis C virus (HCV) infects more than 170 million people worldwide.^1^ The prevalence of HCV is high in China. Approximately 150,000 cases of chronic hepatitis **C** (CHC) were reported in 2010, which are 7 times and 15% higher than those in 2007 and 2009.^2^^,^^3^ Hepatitis-C is transmitted mainly by blood transfusion and blood products infection in the past, which has been prevented by the regulation of the Chinese Government. Therefore, hepatitis c is spread by unsafe injection and iatrogenic infection, including unsterilized dental devices, endoscopes, surgical surgeries, invasive operations and blood dialysis, etc. The anti-HCV positive rate of adults is 3.2%, and that of 1-year-old patients is 2%.

At present, CHC children were treated directly referring to the therapy of adults, i.e. the use of interferon alone or the combined use of pegylated interferon and Ribavirin (RBV).^4^^,^^5^ It has been previously reported that 44%-59% and ~90% of HCV *genotype 1 *as well as *genotype 2 *and 3 children patients acquired sustained virological response (SVR) upon the combined therapy of interferon and RBV.^5^ However, this therapy leads to numerous side effects, such as influenza-like symptoms, reduced leukocytes, anemia, abnormal behaviors, and thyroid function failure.^6^ Recently, some new anti-HCV drugs have been developed. For instance, HCV specifically targeted anti-viral therapy drugs are classified into NS3/4A protease inhibitors, NS5A protease inhibitors, nucleoside and non-nucleoside NS5B polymerase inhibitors, and internal Ribosome entry site (IRES) inhibitors according to their mechanisms.^7^ Nevertheless, this type of drugs has not been allowed to use in China, and the security for children is still undefined as well. Although the combined use of interferon and RBV is given priority in the treatment of HCV, the treatment efficacy still needs improvement.

Single nucleotide polymorphisms (SNPs) near the Interleukin-28B (IL-28B) gene (especially rs12979860 and rs8099917) are highly correlated with the combination of long-acting interferon PEG-IFN-α and RBV.^8^ Meanwhile, the SNPs of IL-28 in different ethnic groups all affect the efficacy of standard anti-HCV treatment, leading to better outcomes in the patients who have protective genes.^9^ The rs12979860 C/T polymorphism is able to predict the spontaneous clearance rate of HCV (*C/C genotype*: 53%; *T/T genotype*: 23.4%). Besides, rs12979860 polymorphism may also predict the spontaneous HCV clearance capacity of children during vertical transmission (especially the rs12979860 *CC genotype* children patients).^10^ Meanwhile, the polymorphism of IL-28B is closely associated with the development of CHC, and *T/T genotype* is more prevalent among cirrhosis patients.^11^ Therefore, understanding IL-28B gene polymorphism is important to predict the treatment therapy of interferon, as well as the spontaneous clearance capacity and histological changes of children patients.

Thereby motivated, this study aimed to understand the distribution characteristics of the SNPs (rs12979860, rs8099917) near IL-28B in Chinese CHC children patients and healthy people, to explore the relationship between SNPs (rs12979860, rs8099917) and spontaneous clearance of HCV in Chinese Han children, and to investigate the correlation between SNPs (rs12979860, rs8099917) and the clinical outcome and histology of CHC children patients.

## METHODS

***Subjects and Grouping: ***CHC children patients hospitalized at the Adolescent Liver Failure Treatment and Research Center, 302 Hospital from March 2011 to March 2012 (277 cases) were selected (Boy: 137 cases; girl: 140 cases; age: 1-17 years old, 4.5 years old in average). Healthy children from May 2011 to March 2012 (150 cases) were selected as the control group (Boy: 78 cases; girl: 72 cases; age: 2-17 years old, 5.2 years old in average**).** Peripheral bloods (2 ml each) were stored in EDTA anticoagulant tubes at -80°C for all patients. The study protocol was evaluated and approved by the ethics committee of 302 Hospital.

***CHC Diagnosis Criteria: ***The Chinese Society of Hepatology, Chinese Medical Association released "Hepatitis c Prevention and Treatment Guidelines" that defines the following patients can be diagnosed as CHC: the patients who have been infected for more than 6 months or have undefined onset dates; the patients, who do not have a history of hepatitis, are diagnoses as CHC by liver histopathological examinations or symptoms, signs, laboratory and imaging results.

***DNA Sampling and Extraction: ***DNA was extracted from 250 μL of the peripheral blood according to the instructions of QIAGEN DNA extraction kit (Hilden, Germany), and the concentrations were detected by UV spectrophotometer (Eppendorf, Germany). The resulting DNA was stored at -80°C.

***IL-28B Genotyping: ***IL-28B was genotyped after the sequencing of rs12979860 and rs8099917. The segments containing rs12979860 andrs8099917 were first amplified by PCR. PCR primers: rs12979860_F CCTCTGCACAGTCTGGGATT and rs12979860_R AGGGACCGCTACGTAAGTCA, rs8099917_F TCACCATCCTCCTCTCATCC and rs8099917_R TGCTGGGCCCTAACTGATAC. They were amplified by GoTaqDNA (Pr omega, USA): denaturation at 95°C for 4 min; denaturation at 95°C for 30 s, annealing at 60°C for 30 s, extension at 72°C for 30s, repetition for 30 cycles, final extension at 72°C for 10 min. The obtained PCR products were sequenced with an ABI 3730 sequencer (Applied Biosysems, Foster City Ca Sa), based on which genotyping was performed.

***HCV Quantification and Genotyping: ***HCV RNA in serum was determined as 15 IU/ml by Cobas Taqman HCV Test (Roche Molecular Diagnostics). HCV genotyping was performed according to the kit instruction (Realchip Biotechnology Co. Ltd, China).

***Histological Examination: ***Live liver tissues were collected by 1-second liver biopsy. The regular liver tissue slices were subjected to HE staining referring to the standard revised by "Viral Hepatitis prevention and treatment program" (10th Infectious and Parasitic Diseases Credits, Chinese Medical Association, Xi'an, 2000). Viral hepatitis cases were graded based on liver histopathological morphologies (inflammatory activity grading: G0-4; fibrosis stage: S0-4).

***Statistical Analysis: ***SPSS12.1 version statistical software was adopted in this paper. T test was used to compare the measurement data of the group of CHC children patients and the healthy experimental group. However, x test was used to analyze the conformity between nucleotide polymorphism in the two groups and the Hardy-Weinberg equilibrium, and in distribution differences and enumeration data of SNP genotypes in the two groups. Pearson χ^2^ test and Fisher exact test are used to analyze gene phenotypes and allele frequencies of locus rs12979860 and rs8099917 in IL-28B gene polymorphism for comparison of the frequency differences between healthy people and patients with hepatitis C. Non-conditional logistic regression analysis was used to adjust ages and genders, and to calculate the odds ratio (OR) and its 95% confidence interval (CI).

**Table-I:** General status and clinical characteristics of CHC children patients

*Clinical characteristic*	
Gender (M/F)	277 (137/140)
Average age	4.5
Infectious reason	
Unsterilized injection history	204 (74%)
Blood transfusion or surgical history	42 (15%)
Undefined	31 (11%)
HCV RNA quantification	
<100 IU/ml	45
10^2^-10^3^ IU/ml	29
10^3^-10^4^ IU/ml	53
10^4^-10^5 ^IU/ml	55
10^5^-10^6^ IU/ml	38
10^6^-10^7^ IU/ml	40
> 10^7^ IU/ml	17
HCV genotype (1b/ 2a)	54/79

**Table-II:** Comparison between rs12979860 and rs8099917 genotypes of the two groups

*SNP*	*Genotype*	*CHC patient (n=277)*	*Control (n=150)*	*P*
rs12979860	C/C	243(87.7%)	127(84.6%)	0.325
	C/T	33(11.9%)	23(15.3%)	0.325
	T/T	1(0.3%)	0	1.0
rs8099917	T/T	241(87.0%)	122(81.3%)	0.117
	T/G	36(13.0%)	28(18.7%)	0.117

**Table-III:** Quantification of initial HCV RNA of CHC spontaneous clearance children patients

*Group*	*Initial HCV RNA (log)*
Spontaneous clearance patient (n=130)	3.01±1.2
Total (n=277)	3.7±1.8*

* P<0.05.

**Table-IV:** Relationship between SNPs (rs12979860, rs8099917) and initial HCV RNA of spontaneous clearance patients

*SNP*	*Genotype*		*Initial HCV RNA quantification (IU/ml)*					
		*Undetected*	*10* ^2^ * - 10* ^3^	*10* ^3^ * - 10* ^4^	*10* ^4^ * - 10* ^5^	*10* ^5^ * - 10* ^6^	*10* ^6^ *- 10* ^7^	*> 10* ^7^
rs12979860	C/C (n=123)	42	20	14	19	14	10	5
	C/T (n=6)	3	1	1	1	0	0	0
rs8099917	T/T (n=126)	44	19	14	20	14	10	4
	T/G (n=4)	1	2	1	0	0	0	5
Spontaneous clearance	n=130	45	21	15	20	14	10	5
CHC	n=277	45	29	53	55	38	40	17

**Table-V:** Relationship between SNPs (rs12979860, rs8099917) and the clinical outcomes of CHC children patients

*SNP*	*Genotype*	*Clinical outcome*			
		*ALT* *(IU/mL)*	*AST* *(IU/mL)*	*TBil* *(μmol/L)*	*HCV RNA (Log)*
rs12979860	C/C	73.9±113.4	62.1±67.2	5.7±2.4	3.7±1.6
	C/T	65.4±58.9	62.5±43.3	6.0±2.8	4.24±1.4
	T/T	41	67	6.8	4
rs8099917	T/T	72.7±112.5	62.1±67.8	4.6±1.6	3.7±1.5
	T/G	73.6±49.6	63.1±41.7	6.09±2.1	4.06±1.9
Total		72.5±108.4	62.2±64.8	5.6±2.5	3.7±1.8

**Table-VI:** Relationship between rs12979860 and rs8099917 genotypes and histological changes

	*Genotype*	*Case Number*	*Inflammatory grade*		*Fibrosis stage*		
			*G1*	*G2*	*S0*	*S1*	*S2*
rs12979860	C/C	n=243	190	53	45	147	51
	C/T	n=33	21	12	19	9	5
	T/T	n=1	1			1	
rs8099917	T/T	n=241	191	50	50	142	49
	T/G	n=36	21	15	14	15	7
Total		n=277	212	65	64	157	56

## RESULTS

***General Status and Clinical Characteristics of CHC Children Patients: ***CHC children patients (277 cases) aging 1-17 years old were selected (Boy: 137 cases; girl: 140 cases). Their average age is 4.55 years old, which does not differ from that of the healthy group (150 cases) significantly. The CHC patients got infected owing to unsterilized injection in small-scale clinics (74%), history of blood transfusion or surgeries (15%), and unknown reasons (11%) (Table-I).

***Distribution Characteristics of SNPs (rs12979860, rs8099917) near IL-28B in Chinese CHC and Healthy Children Patients: ***In the 277 CHC children patients, SNP rs12979860 includes C/C (87.7%), C/T (11.9%) and T/T (only one case) genotypes. In the 150 healthy subjects, SNP rs12979860 includes C/C (84.6%) and C/T (15.3%) genotypes. The genotypes of the two groups do not differ significantly. The rs8099917 genotypes of the two groups also do not differ significantly (Table-II).

***Relationship between SNPs (rs12979860, rs8099917) and HCV Load and Spontaneous Clearance Rate: ***About 47% of the patients were spontaneously cleared (130 cases, log of HCV RNA: 3.01±1.2) (Table-III). Besides, rs12979860 C/C genotype and rs8099917 T/T genotype are closely associated with the clearance of HCV, and the OR values are 7.39 (1.07–50.41) and 14.27 (3.07–108.50). HCV that was low initially was more easily to be cleared (Table-IV).

***Relationship between SNPs (rs12979860, rs8099917) and the Clinical Outcome and Histological Changes of CHC Children Patients: ***The total bilirubin (TBiL) values of CHC children patients were lower than normal, and the values of alanine aminotransferase (ALT) and aspartate aminotransferase (AST) slightly increased. ALT, AST, TBiL and HCV RNA of SNPs rs12979860 and rs8099917 groups do not differ significantly (Table-V).

The histological behavior of CHC patients is G1S1, accounting for 75% of the total cases. In the rs12979860 C/C genotype patients, inflammatory grade G1 and G2 account for 78.2% and 21.8%, and fibrosis stage S0, S1 and S2 account for 18.5%, 60.5% and 21%, respectively. In the C/T genotype patients, inflammatory grade G1 and G2 account for 63.6% and 36.4%, and fibrosis stage S0, S1 and S2 account for 57.6%, 27.3% and 15.1%, respectively. In the rs8099917 T/T genotype patients, inflammatory grade G1 and G2 account for 79.2% and 20.8%, and fibrosis stage S0, S1 and S2 account for 20.7%, 58.9% and 20.4%, respectively. In the T/G genotype patients, inflammatory grade G1 and G2 account for 58.3% and 41.7%, and fibrosis stage S0, S1 and S2 account for 38.9%, 41.7% and 19.4%, respectively (Table-VI).

## DISCUSSION

The spontaneous clearance of HCV infection is low, and 50%-90% of acute hepatitis C patients eventually develop into CHC ones.^12^ IL-28B is a novel interleukin produced by a variety of cells (e.g. peripheral blood monocyte/dendritic cells, Hela cell) induced by viruses or double-stranded RNA. IL-28B is able to combine with a class II cytokine receptor heterodimer consisting of IL-10Rb and IL-28Ra, which resist to various viruses by Jak-STAT signaling pathway. The genetic structure of IL-28B is similar to that of IL-10, but the level of amino acids its codes is close to interferon, which may cooperate with interferon in the inhibition of HCV.^13^ The Duke Center for Public Genomics carried out genome-wide association studies (GWAS) on 1671 subjects and found that the SVR of rs12979860 C/C genotype patients was 2-3 times higher than that of T/T genotype patients after standard therapy. Besides, the distributions of polymorphisms differ by races. The frequencies of C allele in East Asian, European, Spanish and African patients were 95%, 73%, 69% and 41%, which corresponded to the SVR values of 76%, 56%, 51% and 23%, respectively.^14^ Tanaka et al. reported that rs8099917 is also able to predict as rs12979860 does, and the response alleles are T and A.^15^ In this study, rs12979860 C/C genotype and rs8099917 T/T genotype account for 87.7% and 87%, which may be relevant to the higher SVR rates of Chinese Han children. However, the genotypes of the two groups do not differ significantly.

Recently, IL-28B gene polymorphism has been reported to be related with the spontaneous clearance and continuous infection of HCV. Shi et al. also found that rs12979860 was associated with the spontaneous clearance of HCV in Chinese Han patients.^16^ Rao et al. reported that rs80999^17^, rs8105790, rs12980275 and rs10853728 instead of rs12979860 were associated with the spontaneous clearance of HCV.^17^ In the Chinese Han population with HCV infection, the protective genotypes of rs12979860 and rs80999^17^ could evidently enhance the therapeutic effects. Particularly, rs12979860 CC genotype was closely associated with the spontaneous clearance of HCV, and females were more capable of clearing HCV than males did. However, there remain no studies concerning Asian children patients.^18^-^20^

In this study, the spontaneous clearance of HCV in Chinese children patients reached up to 47%, which may be related to the genetic variation of rs12979860 and rs8099917.^21^ Moreover, rs12979860 C/C genotype and rs8099917 T/T genotype occurred frequently because children were included herein instead of adult patients. In addition, the results may also originate from the infection route: 74% of the CHC children patients in the study were infected by unsterilized injection, whereas 61% of adult patients were infected by vein administration.^22^ Besides, rs12979860 C/C genotype and rs8099917 T/T genotype are closely associated with the clearance of HCV, and the OR values are 7.39 (1.07–50.41) and 14.27 (3.07–108.50). Generally, the patients with low baseline viral loads are prone to HCV spontaneous clearance, accompanied by high responses to interferon treatment. Viral load can be used to evaluate the response after interferon treatment as an independent predicting factor.^23^ The high spontaneous clearance of low-viral-load patients may be attributed to the regulation function of the interferon stimulated gene (ISG) expressed in liver cells.^24^

Furthermore, SNP rs12979860 may also be associated with the development of CHC. The rs12979860 T/T genotype is prone to evolving into severe liver fibrosis.^25^ However, most CHC children patients often do not exhibit clinical and biochemical changes (e.g. jaundice). In this study, although the biochemical indices did not change obviously, histological variations (G1S1: 80%), including mild inflammation (e.g. cell invasion, perisnuitis, cholestasis, apoptotic bodies or spotty necrosis) and chronic liver fibrosis, had already occurred. Notably, rs12979860 and rs8099917 genotypes were not correlated with pathological changes, which may be relevant to the few sample number of rs12979860 T/T genotype (only 1 case). A large-scale study is still in need in the future.

## Authors Contributions:

**JH** **:** Designed the protocol.

**FQL a** **nd SS** **Z** **:** Clinical data collection and experiments.

**RR** **W** **:** Prepared the final manuscript.
